# Analysis of Synchronous and Asynchronous In Vitro Infections with Homologous Murine Norovirus Strains Reveals Time-Dependent Viral Interference Effects

**DOI:** 10.3390/v13050823

**Published:** 2021-05-02

**Authors:** Louisa F. Ludwig-Begall, Elisabetta Di Felice, Barbara Toffoli, Chiara Ceci, Barbara Di Martino, Fulvio Marsilio, Axel Mauroy, Etienne Thiry

**Affiliations:** 1 FARAH Research Centre, Faculty of Veterinary Medicine, Veterinary Virology and Animal Viral Diseases, Department of Infectious and Parasitic Diseases, Liège University, 4000 Liège, Belgium; lludwig@uliege.be (L.F.L.-B.); barbaratoffoli83@gmail.com (B.T.); axel.mauroy@favv-afsca.be (A.M.); 2 Department of Diagnosis and Surveillance of Exotic Disease, IZS Istituto Zooprofilattico Sperimentale A&M G. Caporale, 64100 Teramo, Italy; elidifelice@libero.it; 3 Faculty of Veterinary Medicine, Università degli Studi di Teramo, 64100 Teramo, Italy; chiaraceci576@gmail.com (C.C.); bdimartino@unite.it (B.D.M.); fmarsilio@unite.it (F.M.); 4 Staff Direction for Risk Assessment, Control Policy, FASFC, 1000 Brussels, Belgium

**Keywords:** norovirus, murine norovirus, coinfection, superinfection, superinfection exclusion, interference

## Abstract

Viral recombination is a key mechanism in the evolution and diversity of noroviruses. In vivo, synchronous single-cell coinfection by multiple viruses, the ultimate prerequisite to viral recombination, is likely to be a rare event and delayed secondary infections are a more probable occurrence. Here, we determine the effect of a temporal separation of in vitro infections with the two homologous murine norovirus strains MNV-1 WU20 and CW1 on the composition of nascent viral populations. WU20 and CW1 were either synchronously inoculated onto murine macrophage cell monolayers (coinfection) or asynchronously applied (superinfection with varying titres of CW1 at half-hour to 24-h delays). Then, 24 h after initial co-or superinfection, quantification of genomic copy numbers and discriminative screening of plaque picked infectious progeny viruses demonstrated a time-dependent predominance of primary infecting WU20 in the majority of viral progenies. Our results indicate that a time interval from one to two hours onwards between two consecutive norovirus infections allows for the establishment of a barrier that reduces or prevents superinfection.

## 1. Introduction

Human noroviruses (HuNoVs) are recognised as a leading global cause of sporadic and epidemic viral gastroenteritis [1] and account for a global economic burden of $60 billion, over one million hospitalisations, and 200,000 deaths per annum [2,3]. Customarily an acute and self-limiting illness, HuNoV infection can become chronic in the elderly, malnourished, and/or immunocompromised; such patients may experience protracted severe, even lethal, NoV infections and superinfections [4,5,6,7,8]. 

Various HuNoV infection models have yielded valuable insights into the NoV life cycle in recent years [9,10,11,12]. However, many of these experimental systems are technically challenging and as yet lack the degree of robustness required for detailed decipherment. The genetically and biologically closely related murine norovirus (MuNoV), which combines the advantages of available tools for genetic manipulation [13,14], easy in vivo infection of a genetically tractable native host [15], and efficient in vitro propagation [15,16,17], thus remains the main model for NoV in vitro studies.

Human noroviruses and MuNoVs belong to the *Norovirus* genus within the *Caliciviridae* family of small, non-enveloped, positive sense, single-stranded RNA viruses [18,19]. The linear, polyadenlyated 7.4–7.7 kb long HuNoV genome is organised into three open reading frames (ORFs); an additional fourth ORF is described for MuNoVs [20,21]. The 5′ proximal NoV ORF1 encodes a large polyprotein that is co-and post-translationally cleaved into six non-structural viral proteins [22]. ORF2 and ORF3 encode the structural virion components, major and minor capsid proteins, VP1 and VP2, respectively. ORF4, which entirely overlaps the 5′end of ORF2, encodes virulence factor (VF1) [23].

Viral recombination is a key mechanism in the evolution and diversity of NoVs; increasing evidence indicates that recombination shapes NoV pathogenesis and fitness and drives the evolution of emerging strains [24]. Numerous field recombination events, predominantly at a typical ORF1/2 recombination breakpoint [25], have been detected in silico in the *Norovirus* genus [26,27]. In contrast, few experimental data are available concerning NoV recombination under laboratory conditions and the mechanism(s) involved are poorly characterised [26,28,29,30].

We recently identified a set of checkpoints, including their respective drivers and constraints, that must be successfully bypassed for the generation of a viable recombinant NoV [26,31]. Following this, host coinfection, single cell coinfection, and recombination must be accomplished to generate a recombinant NoV RNA. An incipient recombinant viruses must then survive a process of functional selection to be maintained in the viral population [32,33,34,35]. The rise of recombinant viruses resulting from this process is influenced by different factors. In vivo, host coinfection may be dependent on spatial and temporal overlap of strain-distributions. Cell coinfection, the ultimate prerequisite to viral recombination, depends on factors influencing the within-host distribution of viruses to target cells, thereby limiting or increasing the likelihood of cellular coinfections. True coinfection of cells is likely to be a rare event (unless mediated by factors directing synchronous uptake of diverse viruses into both host and cell [36] under natural conditions and delayed secondary infections are a more probable occurrence.

In the event of an asynchronous infection, the uptake of multiple viruses into a single cell is dependent on factors that may limit consecutive entry of more than one virus particle per cell in a process known as superinfection exclusion. Superinfection exclusion is defined as the ability of an established virus to prevent a secondary infection by the same or a closely related virus [37]. The primary infecting virus may render cells refractory to subsequent infection through interference at various stages of the replicative cycle of the secondary invader in a time-dependent manner. Viral pre-and post-entry blocks have been described for a number of RNA viruses [38,39,40,41,42,43,44,45,46]. However, hitherto, NoVs have not been listed amongst them.

Here, we determine the effect of a temporal separation of in vitro infections with the two homologous parental MuNoV strains MNV-1 WU20 and CW1 on the composition of MuNoV populations. A clear advantage of in vitro systems to study viral population dynamics is that they present a well-defined entity containing only viruses and cells. Effects of other factors interfering with cell coinfection (such as the host immune response or microbiome) may thus be discounted.

Our results demonstrate that a time interval from one to two hours onwards between two consecutive NoV infections allows establishment of a barrier that reduces or prevents superinfection; this first demonstration of time-dependent viral interference for NoVs has clear implications for NoV epidemiology, risk assessment, and potentially treatment. 

## 2. Materials and Methods

**Figure 1:**
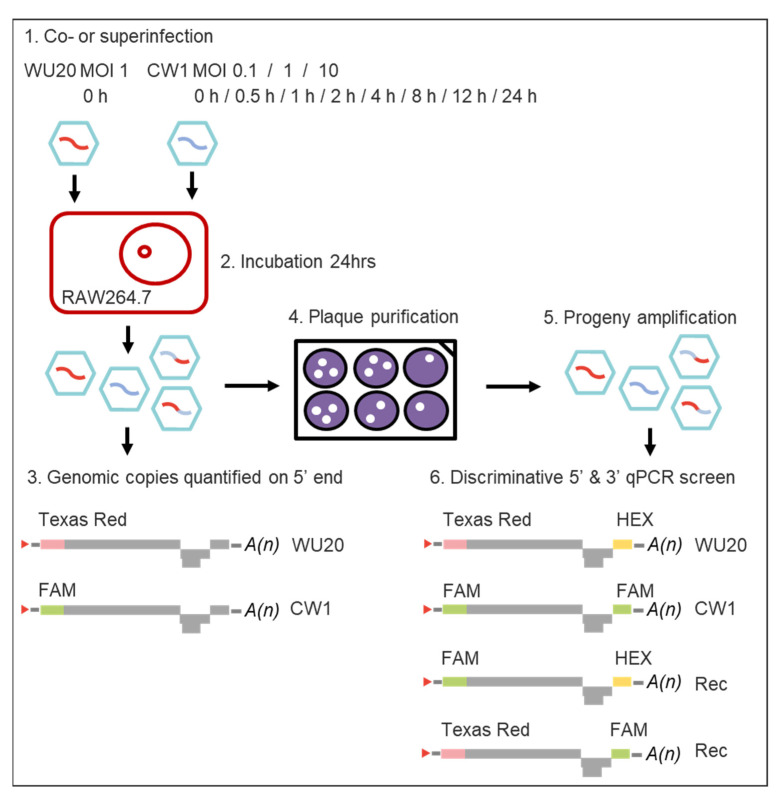
Workflow of the experimental set-up to analyse synchronous and asynchronous in vitro infections with homologous murine norovirus strains MNV-1 WU20 and CW1. MOI = Multiplicity of infection; ORF = Open Reading Frame; qPCR = quantitative polymerase chain reaction.

A graphical overview of all assays is provided in Figure 1.

### 2.1. Viruses and Cells

The murine macrophage cell line RAW264.7 (ATCC TIB-71) was maintained in Dulbecco’s modified Eagle’s medium (DMEMc) (Invitrogen, San Diego, CA, USA, Thermo Fisher Scientific, Waltham, MA, USA) containing 10% heat inactivated foetal calf serum (FCS) (BioWhittaker), 2% of an association of penicillin (5000 SI units mL^−1^) and streptomycin (5 mg ml^−1^) (PS, Invitrogen), and 1% 1 M HEPES buffer (pH 7.6) (Invitrogen) at 37 °C with 5% CO_2_.

Murine NoV isolates MNV-1 CW1 and WU20 (GenBank accession numbers DQ285629 and EU004665.1; 87% nucleotide sequence similarity; previously shown to exhibit highly similar replication kinetics [28,47] were plaque purified and propagated in RAW 264.7 cells as described by Mathijs et al., 2010 [28]. Virus stocks were produced by infection of RAW 264.7 cells at a multiplicity of infection (MOI, expressed as plaque forming units per cell) of 0.05. Two days post-infection, cells and supernatants were harvested and clarified by centrifugation for 20 min at 1000× *g* after three freeze/thaw cycles (−80 °C alternating with 37 °C). Supernatants were purified by ultracentrifugation on a 30% sucrose cushion in a SW28 rotor (Beckman Coulter, Indianapolis, IN, USA) at 23,000 rounds per min for 2 h at 4 °C. Pellets were suspended in 500 µL phosphate-buffered saline (PBS), aliquoted, and frozen at −80 °C. Viral titres were determined via plaque assay for the seventh passage of WU20 and the eighth of CW1 (WU20 P7 and CW1 P8), as described by Hyde et al., 2009 [48]. WU20 P7 and CW1 P8 single-step and multi-step growth curves, performed prior to launching the co-and superinfection experiments described below, exhibited no significant differences in the replication kinetics of the two virus stocks (Supplementary Figure S1). 

### 2.2. Coinfection and Superinfection of RAW264.7 Cells with Murine Noroviruses WU20 and CW1

Monolayers of RAW 264.7 cells were prepared in 24-well plates at a density of 5 × 10^4^ cells per well. Working on ice, each well was infected with WU20 (MOI = 1; confirmed via back-titration). After 1 h, the WU20 inoculums (300 µL) were removed and stored at −80 °C. The cells were washed twice with PBS and were infected with CW1 at various MOIs (0.1; 1; 10; confirmed via back-titration) at delays of 0 min (coinfection), 30 min, and 1, 2, 4, 8, 12, and 24 h (superinfections). For coinfections, CW1 and WU20 inoculums in a final volume of 300 µL were simultaneously added to cells. Cells and virus then remained on ice for 1 h, whereupon the inoculum was removed. For superinfections, CW1 inoculums were asynchronously dispensed onto cells at the appropriate delays, whereupon cells and virus remained on ice for 1 h until removal of the inoculums; the cells were then washed twice with PBS and 300 µL DMEMc were added. Twenty-four hours post co-or superinfection, both cells and supernatants were frozen and stored at −80 °C until further analysis.

### 2.3. Quantification of WU20 and CW1 Genomic Copies in Viral Progenies 24 h Post Co- or Superinfection

RNA extractions were performed with Tri Reagent solution (Ambion, Austin, TX, USA) on 120 µL of co-and superinfection supernatants. Extracted RNA was reverse-transcribed into complementary DNA (cDNA) using an iScript cDNA Synthesis kit (Bio-Rad, Hercules, CA, USA). The extracted and reverse-transcribed cDNA was quantitatively analysed via real time quantitative PCR (qPCR), employing primers to allow discrimination between CW1 and WU20 based upon single nucleotide polymorphisms (SNPs) at the 5′ genomic extremity (amplicon in ORF1, dubbed region 1), as described by Mathijs et al. 2010. Primers and probes used in the quantification of genomic copies correspond to those listed in Supplementary Table S1 as published by Mathijs et al., 2010 [28].

Quantifications were performed as previously described by Mauroy et al. (2012) [49]; for generation of the standard curve, region 1 amplicons were amplified for both CW1 and WU20, then cloned into a pGEMt-Easy vector (Promega, Madison, WI, USA) and sequenced. Both CW1-region 1 and WU20-region 1 plasmids were in vitro transcribed with the Ribomax kit (Promega) following manufacturer’s instructions. Briefly, *Spe*I-linearised and purified plasmids were transcribed with T7 RNA polymerase, treated with DNAse, and quantified via spectrophotometer. Genomic copy numbers of transcribed RNA were deduced and serial ten-fold dilutions were prepared with ultrapure RNAse free water (Invitrogen). Aliquots of the master stock were stored at −80 °C and measured before dilution and use. Final results were normalised using transcripts of the housekeeping gene glyceraldehyde 3-phosphate dehydrogenase (GAPDH) (Barber et al. 2005). A 5 µL qPCR mix (technical duplicates) was set up by adding 1 µL of cDNA to 2.50 µL of iQ supermix, 0.1 µL of both GAPDH-forward and -reverse primers (100 nM final concentration), 0.2 µL of the GAPDH-probe (200 nM final concentration) and 1.1 µL of nuclease free water. Cycling conditions included an initial 5-min denaturation at 95 °C followed by 38 cycles of 10 s at 95 °C and 40 s at 60 °C.

### 2.4. Isolation and Screening of Infectious Progeny Viruses

Cells and supernatants from the co- and superinfection step were frozen and thawed once and then utilised as inoculums in a plaque assay for purification of infectious progeny viruses following the method described by Hyde et al. 2009 with slight modifications [48]. Briefly, RAW 264.7 monolayers, cultured in six-well plates (2 × 10^6^ RAW264.7 cells/well) were inoculated at room temperature with 1 mL of serial dilutions of virus-containing culture fluids of the co- and superinfection assays. After 1 h, inoculums were removed and cells were overlaid with 2 mL of medium containing 70% DMEM-Glutamax (4.5 g glucose l-1 and 15 mM sodium hydrogen carbonate), 2.5% FCS, 2% PS, 1% HEPES and 0.7% SeaPlaque agarose (Lonza, Basel, Switzerland) per well. After 48 h of incubation (37 °C, 5% CO_2_), 36 individual plaques were randomly selected per condition. Infected cells from the plaque margins were picked with a needle under a microscope and were diluted into fresh DMEMc before propagation by inoculation onto RAW 264.7 cells grown in 24-well plates. After 72 h, supernatants were collected and frozen at −80 °C until further analysis.

Following RNA extraction and reverse transcription, cDNAs of individual plaque-purified virus progenies were analysed via two parallel real time PCR runs employing two pairs of primers to allow discrimination between CW1, WU20 (and recombinant) signals based upon single nucleotide polymorphisms (SNPs) at both genomic extremities (ORF1 and ORF3, dubbed regions 1 and 5, respectively) as described by Mathijs et al. 2010 [28]. Five µl reactions were carried out with iQ supermix. Primers and probes for this TaqMan-based discriminative qPCR correspond to those listed in Supplementary Table S1 as published by Mathijs et al. 2010 [28]. 

In the case of ambiguous signals originating from mixed virus populations, an additional quick screen was performed via Sanger sequencing of the ORF1/2 overlap (base pairs 4864 to 5298 in MNV-1 CW1), this to exclude the presence of potential recombinants or PCR chimeras from interfering with later calculations of WU20 to CW1 infectious virus ratios. 

## 3. Results

### 3.1. Absolute and Relative Quantification of Genomic Copies Reveals Skewed WU20 and CW1 Distributions and a WU20 Dominance in Most Viral Progenies 24 h Post Co- or Superinfection

**Figure 2:**
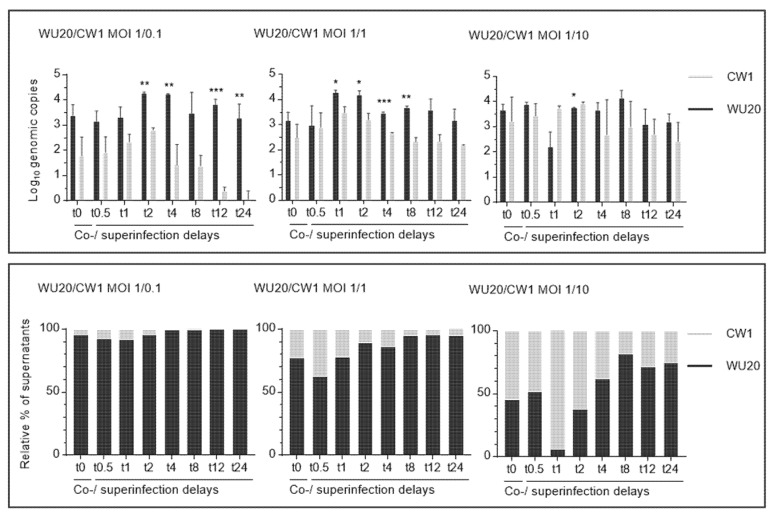
Genomic quantification on 5′ genome ends establishing raw genomic copy numbers (top) and relative proportions of mean genomic copies (below) of co- or superinfecting murine noroviruses MNV-1 WU20 and CW1 in viral progenies 24 h post co- or superinfection. Genomic copy numbers and their relative proportions resulting from one co-infection (t0) and seven asynchronous infections (primary infection: WU20; superinfection at half-hour to 24-h delays (t0.5 to t24): CW1) are shown. Varying multiplicities of infection (MOI) were analysed; the MOI of primary infecting WU20 remained stable at 1 throughout all assays while the MOI of superinfecting CW1 varied between 0.1 (left panels), 1 (middle panels), and 10 (right panels). Black bars represent WU20, grey bars represent CW1. Differences in yield between mean WU20 and CW1 genome copies were analysed using GraphPad Prism 7 (Graph-Pad Software) and *p* values were determined using two-sided unpaired-sample t tests, where *** *p* ≤ 0.001, ** *p* ≤ 0.01, and * *p* ≤ 0.05.

To quantitatively assess viral progeny distributions 24 h after initial co-or superinfection, MNV-1 WU20 and CW1 genomic copy numbers were inferred from the cycle threshold (Ct) values of the qPCR reactions and normalised against GAPDH Ct values. This genomic quantification (5′ region 1 amplicon) revealed WU20 absolute genomic copy numbers, averaging 3.55 (±0.57) log_10_ genomic copies over all measured time points, to be higher than those of CW1 in all but four of the resulting 24 viral progenies. Only short superinfection delays (t0 h, 0.5 h, 1 h, 2 h) with a WU20 to CW1 starting ratio of one to ten resulted in CW1 genomic copy numbers significantly higher than or equal to those of WU20 24 h post co- or superinfection at 3.23 (±0.96), 3.44 (±0.48), 3.74 (±0.09), and 3.93 (±0.06) log_10_, respectively (Figure 2, top panels). 

These absolute genomic copy numbers translate into relative ratios of genomic copies that reflect a disproportionate WU20 dominance within the majority of viral populations (Figure 2, bottom panels). A WU20 to CW1 starting MOI ratio of 1 to 0.1 (expected to yield 90% WU20 and 10% CW1 genome copies upon qPCR analysis of the viral population) yielded mean WU20 genomic copy numbers of 3.40 (±0.43), 3.17 (±0.34), 3.33 (±0.40), and 4.27 (±0.05) log_10_ (accounting for 95.98%, 92.46%, 91.74%, and 95.73% of the population) 24 h after either coinfection (t0) or superinfections with delays of half an hour (t0.5) to two hours (t2). From a superinfection delay of four hours (t4) onwards, WU20 mean genomic copy numbers ranging from 3.28 (±0.56) to 4.24 (±0.01) log_10_ (99.43 to 99.96% of the population) are juxtaposed against CW1 values of 1.43 (±0.81) to 0.07 (±0.32) log_10_.

At an equal WU20 to CW1 starting MOI of 1 (expected yield to 50% WU20 and 50% CW1 genome copies), 3.17 (±0.33) log_10_ (77.30%) WU20 to 2.49 (±0.54) log_10_ (22.69%) CW1 (t0) and 2.98 (±0.78) log_10_ (62.64%) WU20 to 2.89 (±0.59) log_10_ (37.36%) CW1 ratios (t0.5), are succeeded by a marked increase of the WU20 proportion, covering 4.27 (±0.11) (77.63%) (t1), 4.17 (±0.18) (89.26%) (t2), and 3.37 (±0.04) log_10_ (86.01%) (t4), and then reaching values of over 3.16 (±0.47) log_10_ (95%) from t8 onwards, while CW1 values are consistently at least one order of magnitude lower and never surpass 2.67 (±0.02) log_10_ from t4 onwards.

A WU20 to CW1 starting MOI ratio of 1 to 10 (expected to yield 10% WU20 and 90% CW1 genome copies) resulted in 3.66 (±0.24) log_10_ to 3.23 (±0.96) log_10_ and 3.88 (±0.09) to 3.44 (±0.48) log_10_ WU20 to CW1 genome copies at t0 and t0.5, respectively (roughly 50–50 ratios), fulfilled expectations with 2.2 (±0.59) log_10_ WU20 to 3.74 (±0.09) log_10_ CW1 genome copies (6.16% WU20 to 93.84% CW1) at t1, after which WU20 genome copy numbers progressively increased to 3.76 (±0.01), 3.68 (±0.29), 4.16 (±0.3), 3.09 (±0.63), and 3.19 (±0.32) log_10_ (accounting for 37.85%, 61.66%, 81.67%, 71.20%, and 74.58% of the population) at t2, t4, t8, t12, and t24, respectively. CW1 genome copy numbers correspondingly decreased. 

### 3.2. Molecular Screening on Picked Lysis Plaques Demonstrates a WU20 Predominance in the Majority of Infectious Viral Progenies 

To isolate and screen infectious progeny viruses present within the various viral populations 24 h after initial co-or superinfection, 36 viral plaques per condition were picked from a plaque assay, further propagated in RAW246.7 cells, and then analysed in parallel duplex qPCR runs to discriminate between MNV-1 CW1 and WU20 (as well as possible recombinant viruses) based on 5′ and 3′ SNPs. In three cases, additional ORF1/2 screening confirmed sequence kinship to either WU20 or CW1.

**Figure 3:**
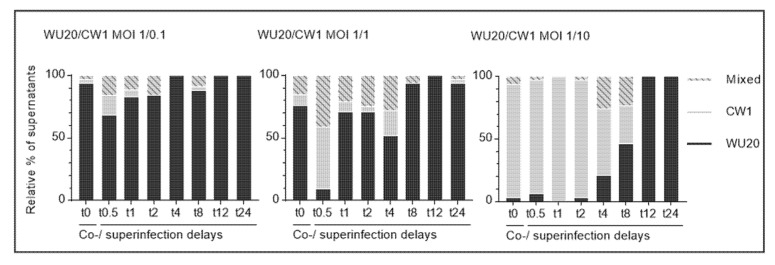
Relative proportions of viable co- or superinfecting murine noroviruses MNV-1 WU20 and CW1 after plaque purification and amplification. Black bars show the proportion of WU20, grey bars show the proportion of CW1, and striped bars indicate mixed signals of both WU20 and CW1 in viral progenies amplified from 36 plaques per condition. One coinfection (t0) and seven asynchronous infections (primary infection: WU20; superinfection at half-hour to 24-h delays (t0.5 to t24): CW1) and varying multiplicities of infection (MOI) were analysed; the MOI of primary infecting WU20 remained stable at 1 throughout all assays while the MOI of superinfecting CW1 varied between 0.1 (left panel), 1 (middle panel), and 10 (right panel)).

Overall, the previously observed WU20 dominance, particularly following longer CW1 superinfection delays, is mirrored in the proportions of plaque picked infectious viruses (Figure 3). Thus, a WU20 to CW1 starting MOI ratio of 1 to 0.1 (expected to yield infectious progeny virus proportions of 90% WU20 to 10% CW1) yielded 94%, 68%, 83%, 84%, 100%, 88%, 100%, and 100% WU20 24 h after coinfection (t0) or superinfection delays of half an hour (t0.5) to 24 h (t24), respectively. Pure CW1 fractions are seen to account for 3%, 16%, and 6% of infectious viral populations at t0, t0.5, and t1. However, with the exception of the eight-hour superinfection delay (3% CW1 at t8), CW1 is not represented in infectious virus progenies from t2 onwards. Mixed WU20 and CW1 progenies make up the remaining fractions of the various populations. 

At an equal WU20 to CW1 starting ratio (expected to yield balanced infectious WU20 and CW1 proportions), initial 76% WU20 to 9% CW1 (plus 15% mixed) and 9% WU20 to 50% CW1 (plus 41% mixed) ratios at t0 and t0.5 are succeeded by a marked increase of the WU20 proportion. WU20 thus accounts for 67%, 71%, and 52% of infectious viral progenies at t1, t2, and t4, and consistently reaches values of over 94% from an eight-hour superinfection delay (t8) onwards. CW1 and mixed progeny proportions correspondingly decrease following the one-hour superinfection delay (t1). 

A WU20 to CW1 starting MOI ratio of 1 to 10 (expected to yield infectious progeny virus proportions of 10% WU20 to 90% CW1) resulted in WU20 proportions of 3% following coinfection (t0) and 6%, 0%, and 3% following early superinfection delays (t0.5 to t2). From t4 onwards, WU20 quantities are seen to progressively increase, accounting for 21% (t4), 46% (t8), and 100% (t12 and t24) of infectious virus progenies. 

## 4. Discussion

Viral recombination has been identified as a key mechanism shaping the evolution and diversity of NoVs [24,26,27]. In contrast to an abundance of field data, few experimental data are available concerning NoV recombination and the mechanism(s) involved remain poorly characterised [26,28,29,30,50]. An incremental step in the generation of any recombinant viral RNA and consequently any viable recombinant virus is the successful simultaneous infection of a single cell by (a minimum of) two viruses [32,33,34,35]. Under natural conditions, various environmental, host, and virus factors may influence the probability of synchronous coinfections and may determine the delay or even the absolute achievability of asynchronous cellular superinfections. Superinfection exclusion, whereby a primary infecting virus may render cells refractory to subsequent infection through interference at various stages of the replicative cycle of the secondary invader [37], is a typically virus-mediated process. Viral pre-and post-entry blocks have been described for a number of RNA viruses [38,39,40,41,42,43,44,45]. Hitherto, NoVs have not been listed amongst them.

Here, we determined the effect of a temporal separation of in vitro infections with the two homologous MuNoV strains MNV-1 WU20 and CW1 on the composition of nascent MuNoV populations. In utilising an in vitro system, we excluded both environmental and host influences and were thus able to examine only those effects mediated by the viruses themselves.

Subsequent to initial WU20 and CW1 cell coinfections or superinfections with half-hour- to 24 h-delays and varying input MOIs (1:0.1; 1:1; 1:10), followed by a 24-h propagation step, individual viral progeny distributions were analysed via qPCR. This quantitative analysis revealed a disproportionate dominance of primary infecting WU20 genomic copies in the majority of resulting viral progenies. While the WU20 dominance appeared to be near-independent of the input MOI ratios of the two viruses (and indeed skewed expected genomic copy ratios throughout), it was markedly time-dependent; increasing CW1 superinfection delays from one to two hours onwards were associated directly with increasing WU20 genome copy fractions. While primary infecting WU20 is expected to have undergone a round of replication before addition of CW1 at superinfection delays of more than eight hours (thus inherently tipping the balance of virus ratios in favour of WU20), input and expected ratios deviate significantly even at earlier time points where this effect cannot serve to explain the observed WU20 dominance.

Interestingly, the way in which higher-than-expected WU20 genomic copy numbers skewed expected genomic copy ratios even after coinfections or short superinfection delays may hint at the mechanism of the pronounced dominance following longer delays. Where input MOIs of 1:0.1, 1:1, and 1:10 were expected to yield WU20 to CW1 genomic copy ratios of 90% to 10%, 50% to 50%, and 10% to 90% following coinfection, these expectations were frustrated in the face of 3.40 (±0.43) to 1.79 (±0.75), 3.17 (±0.33) to 2.49 (±0.54), and 3.66 (±0.24) to 3.23 (±0.96) log_10_ WU20 to CW1 genomic copy proportions. Vacillating levels of infectious virus and genomic copies have previously been associated with the presence of defective interfering (DI) RNAs or DI particles within NoV populations [51]. DI RNAs or particles, deleterious virus-like by-products of error-prone RNA virus replication, are known interfere with standard virus particles by competing for resources [52,53]. DI RNAs may also play a role in mediating superinfection exclusion by induction of RNA silencing and the homology-dependent degradation of incoming RNA molecules [54]. In this context, it is conceivable that WU20 DI RNAs within the population (necessarily included in the quantitative analysis of genome copies since the qPCR assay does not distinguish between DI RNAs or DI particles and whole (infectious) viral genomes) were recognised by the cellular RNA silencing machinery and served to guide degradation of incoming CW1 RNA sequences, this particularly following longer superinfection delays.

Relative proportions of infectious viruses isolated from viral progenies following coinfection (t0) or short superinfection delays of up to two hours (t0.5 to t2), support a possible role of WU20 DI RNAs. Thus, e.g., coinfection with a one to ten WU20 to CW1 MOI ratio resulted in skewed genomic copy ratios of 45.57% WU20 to 54.43% CW1, but translated into infectious virus ratios of 3% WU20 and 91% CW1 (plus 6% mixed). Following asynchronous infection with longer delays (t4 to t24), a time-dependent WU20 dominance and corresponding CW1 decrease is evident within infectious virus progenies. Mixed populations registered subsequent to two-hour superinfection delays may indicate that that the barrier is established progressively and is, initially, not strong enough to completely repel superinfecting CW1, especially in the face of high input titres. 

Taken together, these results demonstrate that a time interval from one to two hours onwards between two consecutive in vitro MuNoV infections allows establishment of a barrier that progressively reduces or prevents superinfection. While viral interference, or superinfection exclusion, has hitherto not been described for NoVs, it is well documented for other positive sense, single-stranded RNA viruses, such as hepatitis C-, bovine viral diarrhoea-, and West Nile virus and may be established within 30 min to several hours of primary infection [43,44,55,56,57].

In future investigations it will be interesting to leverage population-level deep sequencing to analyse how the viral interference effects pinpointed here may influence the generation of NoV RNA recombinants (and thus ultimately influence the chances of recombinant virus generation under the application of selective pressures). Further work should also focus on the mechanism of NoV interference (pre-or post-entry mode of action analysis) and will investigate whether the observed block can be overcome by superinfecting viruses.

Understanding the influence that viral interference may have on NoV population dynamics has clear implications for NoV epidemiology and risk assessment. The phenomenon is thought to decrease the evolution of drug resistance and immune escape by limiting population variability and virus recombination [55]. Identifying where it plays a role and also where and how it may be overcome in the field by superinfecting variants are also important in the context of treating NoV infections.

## Supplementary Materials

The following data are available online at https://www.mdpi.com/article/10.3390/v13050823/s1, Figure S1: Growth curves for MNV-1 WU20 and CW1 at low and high multiplicities of infection (MOI).
