# Supplementary figures and images for: Analysis of Synchronous and Asynchronous In Vitro Infections with Homologous Murine Norovirus Strains Reveals Time-Dependent Viral Interference Effects
Source: Viruses. 2021 May 2;13(5):823. doi: 10.3390/v13050823 (PMC8147416; doi:10.3390/v13050823)

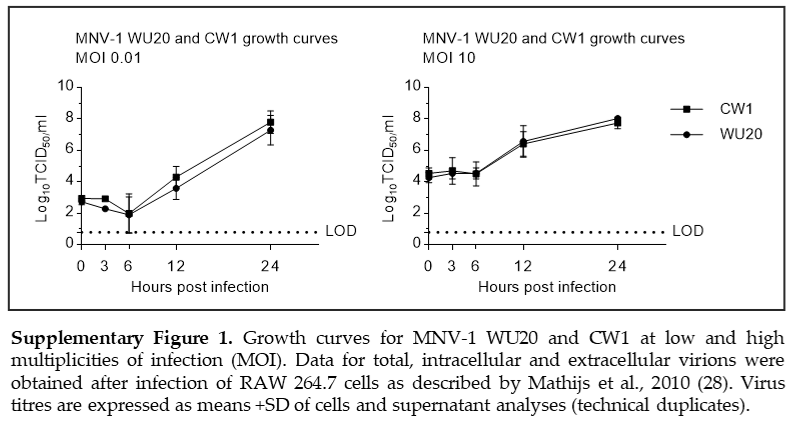

Supplement: Supplementary file 1 [file viruses-13-00823-s001.zip › viruses-1173332.png]
